# Supplementary material for: Identity construction in the very old: A qualitative narrative study
Source: PLoS One. 2022 Dec 15;17(12):e0279098. doi: 10.1371/journal.pone.0279098 (PMC9754203; doi:10.1371/journal.pone.0279098)
Supplement: Attachment — Submitted filename: PONE-D-22-09522 review.pdf [file pone.0279098.s001.pdf]

Thanks for the opportunity to review this manuscript, which I enjoyed reading and I think can make a valuable contribution to the literature.

To situate my perspective as a reviewer without giving my identity away, I'm a person who studies the life course using mixed methods. My particular interest as they touch this subject is in the health and aging of returned soldiers. So I read the manuscript with somewhat of an eye to what we can learn from studying this particular group, and how that might inform how we study other groups of aging people.

The valuable contribution of this manuscript is the engagement with a rarely interviewed demographic, and I think it will be of interest to people because of that. The paper makes its methodological approach clear with the use of Positioning Theory.

There are a few ways in which I think the paper could, with minor modifications, be of interest to an even wider audience.

1) More clearly articulating general insights we can learn from the study of identity work in this oldest old group. The themes articulated by the elderly in this specific setting are likely to be echoed elsewhere, though with perhaps different inflections or emphases reflecting the social setting of aging adults in different societies. Perhaps in the discussion the authors could address social situations which may be isomorphic to the oldest old. One analogy that comes to mind is how different generations of workers in workplaces relate to each other.
    a) Related to this, the authors could be more explicit on why 95+ is distinctly different than merely being in one's late 80s or early 90s (I looked up UK life tables as I was reading this, and it really emphasizes how selective this group is compared to the mere 86 year olds. https://www.ons.gov.uk/peoplepopulationandcommunity/birthsdeathsandmarriages/lifeexpectancies/datasets/nationallifetablesunitedkingdomreferencetables)
2) Implicit in the manuscript as it stands is that the oldest old are doing a lot of relationship work. As currently framed, the paper emphasizes the individual work and presentation of self. I would encourage the authors to be more explicit about the common thread in these discussions of re-negotiating relationships with e.g. adult children and caregivers. I found it curious that there was no citation to, or engagement, with Erving Goffman and work that draws on his insights about the presentation of self in everyday life. I'd encourage the authors to make that connection to sociological work on identity and relationship re-formation more clearly.
3) The specific context of the study is stated, but what it means to be 95+ in the northeast of England (obviously varies!) in the 2010s and early 2020s isn't described. Specifically here, I'd like the authors to be clearer on the life course and cohort aspects of their sample. Particularly for readers from other cultures, or for readers from the same culture reading this later, what does it mean to have been born in the 1920s (approximately) and survived 95+ years. In broad strokes, some of the likely contours of family life, work, and

gender relations that this group would have encountered will be helpful to contextualize the responses.

4) The manuscript is relatively silent about gender differences, except when discussing appearance. Given the differences in survival between women and men to these ages, this is a little puzzling. Were there differences between men and women how participants expressed identities? Perhaps there weren't, and that would be interesting in itself. But I think it bears being more explicit that in other aspects than appearance the ideas expressed by men and women were similar. This would make sense, just given the rarity of survival to these ages.
5) The manuscript emphasizes the relationship work these subjects do with people younger than themselves. did the subjects discuss their relationships with similarly aged peers. Understandably these may become less important as the surviving peer group shrinks dramatically, particularly for men.

Thanks for the opportunity to review this manuscript. I found it stimulating, and think that the paper will be of interest to a broad audience. The methodological discussion of interviewing with this group was also useful in its frank acknowledgement of the challenges.
